# Metabolic engineering of yeast to produce fatty acid-derived biofuels: bottlenecks and solutions

**DOI:** 10.3389/fmicb.2015.00554

**Published:** 2015-06-08

**Authors:** Jiayuan Sheng, Xueyang Feng

**Affiliations:** Biomolecular Engineering Lab, Department of Biological Systems Engineering, Virginia Polytechnic Institute and State UniversityBlacksburg, VA, USA

**Keywords:** fatty acid biosynthesis, *S. cerevisiae*, *Y. lipolytica*, acetyl-CoA, metabolic regulation

## Abstract

Fatty acid-derived biofuels can be a better solution than bioethanol to replace petroleum fuel, since they have similar energy content and combustion properties as current transportation fuels. The environmentally friendly microbial fermentation process has been used to synthesize advanced biofuels from renewable feedstock. Due to their robustness as well as the high tolerance to fermentation inhibitors and phage contamination, yeast strains such as *Saccharomyces cerevisiae* and *Yarrowia lipolytica* have attracted tremendous attention in recent studies regarding the production of fatty acid-derived biofuels, including fatty acids, fatty acid ethyl esters, fatty alcohols, and fatty alkanes. However, the native yeast strains cannot produce fatty acids and fatty acid-derived biofuels in large quantities. To this end, we have summarized recent publications in this review on metabolic engineering of yeast strains to improve the production of fatty acid-derived biofuels, identified the bottlenecks that limit the productivity of biofuels, and categorized the appropriate approaches to overcome these obstacles.

## Introduction

The industry of fuel production continues to grow at an increasing rate each year. Currently, around one third of fuels are derived from petroleum feedstock worldwide while the rest are from coal, natural gas, nuclear energy, hydro–electricity and renewables (BP, 2014). Around 80% of the liquid transportation fuels are derived from petroleum (Floudas et al., 2012). The rapidly depleting petroleum reservoirs and growing environmental concerns on using petroleum feedstock make it increasingly important to shift to a renewable-based industry (Zhang et al., 2011a). Among various forms of renewable energy, microbial production of biofuels, despite drawbacks such as low benefit-cost ratio and scale-up issues, stands as a promising replacement of petroleum fuel since they are environmentally and economically friendly. First generation biofuels, such as bioethanol fermented from corn and biodiesel esterified from edible vegetable oils or animal fats, cover over 90% of the current biofuel market (Antoni et al., 2007). This kind of replacement of petroleum fuel would require occupying huge amounts of farmland for biofuel production, which would challenge the world food supply and cause a series of economic problems (Peralta-Yahya et al., 2012). Therefore, second generation biofuels, such as the advanced biofuels, can be a better solution, since they can be produced from low-cost agricultural byproducts, including wheat straw, forest waste, and energy crops (e.g., switchgrass) (Zhang et al., 2011a). Proposed advanced fuels include butanol (Connor and Liao, 2009; Yan and Liao, 2009), isopentanol (Zhang et al., 2011a), terpenes (Peralta-Yahya et al., 2011; Zhang et al., 2014), fatty acid ethyl esters (FAEEs) (Fukuda et al., 2001; Steen et al., 2010), and alkanes (Schirmer et al., 2010; Peralta-Yahya et al., 2012), which have very similar energy content, storage and transportation properties, and combustion properties as those in current transportation fuels, allowing these advanced biofuels to be directly used in existing gasoline, diesel, and jet engines (Hamelinck and Faaij, 2006; Peralta-Yahya et al., 2012).

Among the advanced biofuels, fatty acid-derived compounds, including free fatty acids (FFAs), fatty alcohols, FAEEs, or fatty acid methyl esters (FAMEs), and fatty alkane/alkenes, are of particular interest, since they fulfill a role as platform molecules of a cluster of important fuels. FFAs serve as precursors to soaps, surfactants, and lubricants (Tee et al., 2014) and can be converted to fatty alcohols, FAEEs and FAMEs. Fatty alcohols are widely used in detergents, skin care products, cosmetics, and medicines, and are also considered to be potential biofuels (Liu et al., 2014). FAEE and FAME are excellent diesel fuel replacements due to their low water solubility and high energy density, and are suitable for microbial production because of their low toxicity to host cells (Zhang et al., 2012). Alkanes, observed throughout nature, are the major constituents of gasoline, diesel, and jet fuel (Kissin, 1987). The natural fatty acid market was valued at $7.2 billion in 2011 and the total market value is expected to reach $13 billion in 2017 after increasing at a 5-year compound annual growth rate (CAGR) of 13.6% (BCC Research LLC, 2013). The applications of fatty acid derivatives had a value of nearly $3.3 billion in 2012, and are expected to have a value of $6.5 billion in 2017 (BCC Research LLC, 2013).

The microbial production of fatty acid-derived biofuels have recently been achieved in many industrial applications (Trotter, 2001; Strijbis and Distel, 2010; Lennen and Pfleger, 2013; Jones et al., 2014; Lee et al., 2014; Beld et al., 2015; Jia et al., 2015; Pasztor et al., 2015; Pfleger et al., 2015). There have been numerous literatures focusing on the overproduction of fatty acid-derived biofuels in the past 5 years, most of which have used *Escherichia coli* as the host (Steen et al., 2010; Zhang et al., 2011b; Youngquist et al., 2012; Xu et al., 2013; Jones et al., 2014; Chu et al., 2015; Clomburg et al., 2015; Liu et al., 2015a; Tai et al., 2015). Although the number of literatures published on fatty acid production using yeast such as *S. cerevisiae* and *Yarrowia lipolytica* is far more limited than that of *E. coli*, yeast has its own advantages which make it an appealing host. First, the synthetic route of fatty acids is more direct and shorter than that of *E. coli*, which allows more efficient conversion of carbon substrate to fatty acids and fatty acid-derived biofuels (Zhang et al., 2011a; Runguphan and Keasling, 2014). Also, yeast is a robust industrial organism that can grow under low pH levels and various harsh fermentation conditions and is free of phage contamination (Hong and Nielsen, 2012; Runguphan and Keasling, 2014). Many of the yeast strains have been fully sequenced and well characterized for their metabolic pathways with the availability of a cluster of genetic tools (Hong and Nielsen, 2012). Recently, there has been an increasing interest in developing yeast as a cell factory for fatty acid-derived biofuel production. However, engineering yeast to produce fatty acid-derived biofuels also faces many challenges. In fact, the highest titer of fatty acids, fatty alcohol and FAEEs could only reach 2.2, 1.1, and 0.52 g/L, respectively, in yeast (Table 1), which is even lower than that of *E. coli* (8.6, 1.7, and 11 g/L, respectively). Long-chain alkane production in yeast has not been successfully achieved until a very recent research breakthrough in metabolic engineering of *S. cerevisiae*, which, however, can only produce 13.5 μg/L heptadecane (Buijs et al., 2014).

**Table 1:** **Comparison of the productivity of fatty acid-derived biofuels between *E. coli* and *S. cerevisiae***.

	***E. coli***		***S. cerevisiae***	
	**Titer (g/L)**	**References**	**Titer (g/L)**	**References**
Fatty acids	8.6	Xu et al., 2013	2.2	Cardenas and Da Silva, 2014
Fatty alcohols	1.95	Cao et al., 2015	1.1	Feng et al., 2014
FAEEs	11	Elbahloul and Steinbuchel, 2010	0.52	Yu et al., 2012
Fatty alkanes	0.58	Choi and Lee, 2013	0.0001^*^	Buijs et al., 2014

* *The data was calculated based on the assumption of biomass was 5 g/L. The original data was 22.0 ± 1.4 μg/g DCW*.

In this review, we summarized the research achievements that have been accomplished in the past 5 years on engineering yeast (mostly *S. cerevisiae* and *Y. lipolytica*) to produce fatty acid-derived biofuels (Table 2). We focused on discussing the limitations that have been discovered by many of the researchers in the area of metabolic engineering when tuning the fatty acid metabolism in yeast strains. We then summarized the synthetic and systems biology approaches to overcome these difficulties. We also identified several potential issues that have not yet been well addressed when modifying yeast metabolism for improved production of fatty acid-derived biofuels.

**Table 2:** **Metabolic engineering of yeast to improve the production of fatty acid-derived biofuels**.

**Strategies**	**Target**	**Strain**	**Genetic manipulation**	**Titer/achievement**	**References**
Improving precursor supplement	FFA (C16: 66.3%, C18: 21.1%)	BY4727	Overexpression of TesA, *ACC1, FAS1*, and *FAS2*	0.4 g/L	Runguphan and Keasling, 2014
	FFA (C16, C18)	BY4741	Overexpression of *Mus musculus ACOT5*	0.493 g/L	Chen et al., 2014
	FFA (C16, C18)	BY4741	Overexpression of *Mus musculus* ACL. Deletion of *IDH1* and *IDH2*	0.13 g/L^*^	Tang et al., 2013
	FFA (C16, C18)	YPH499	Deletion of *FAA1* and *ADH1*	0.14 g/L	Li et al., 2014
	FFA (C16, C18)	CEN.PK2	Overexpression of the reversed β-oxidation pathway and SeAcsL641P. Deletion of *ADH1, ADH4, GPD1*, and *GPD2*	0.011 g/L	Lian and Zhao, 2015
	TAL	BY4741	Overexpression of the *Gerbera hybrid* 2-pyrone synthase (2-PS)	2.2 g/L	Cardenas and Da Silva, 2014
	Fatty alcohol (C16: 91.1%; C18: 8.9%)	BY4742	Overexpression of mouse *FAR*, *ACC1, FAS1, FAS2*	0.086 g/L	Runguphan and Keasling, 2014
	FAEE (C16, C18)	BY4742	Overexpression of Ab*WS, ACC1, FAS1*, and *FAS2*. Deletion of *POX1*	0.005 g/L	Runguphan and Keasling, 2014
	FAEE (C16, C18)	BY4741	Overexpression of *WS2*. Deletion of *FAA2, ACB1, PXA2*	0.025 g/L	Thompson and Trinh, 2014
	FAEE (C16, C18)	CEN.PK113	Overexpression of *WS2*. Deletion of *ARE1, DGA1, ARE2, LRO1*, and *POX1*	0.017 g/L	Valle-Rodríguez et al., 2014
	FAEE (N/A)	CEN.PK113	Overexpression of *WS2, ADH2, ALD6*, and Se*Acs*^L641P^	0.002 g/L^*^	de Jong et al., 2014
	FAEE (C16, C18)	CEN.PK113	Overexpression of WS2, ACB1, and GAPN	0.048 g/L	Shi et al., 2014a
	FAEE (C16, C18)	CEN.PK113	Overexpression of *WS2* and phosphoketolase pathway	0.026 g/L^*^	de Jong et al., 2014
	FAEE (C4–C10)	CEN.PK113	Overexpression of the reversed β-oxidation pathway and *EEB1* or *EHT1*	0.75 g/L	Lian and Zhao, 2015
	FAEE (Medium chain)	CEN.PK2	Overexpression of Ab*WS, GUP1, GCY1*, and *DAK1*. Deletion of *FPS1*, and *GPD2*	0.52 g/L	Yu et al., 2012
	Alkane (Very long chain)	INVSc1	Overexpression of *SUR4*^F262A,K266L^ and *A. thaliana CER1* and *CER3*	Trace	Bernard et al., 2012
Improving cofactor supply	3-HP	CEN.PK113-11C	Overexpression of *Streptococcus mutans* GAPN and *Kluyveromyces lactis* GAPDH	0.463 g/L	Chen et al., 2014a
	Fatty alcohol (C16, C18)	BY4727	Introduce a malic enzyme *Mortierella alpina* ME	0.098 g/L	Runguphan and Keasling, 2014
	Alkane Long chain (C13, C15, C17)	CEN.PK113-11C	Overexpression of *Se*FAR and *Se*FADO. Deletion of *HFD1*	1.1 × 10^−4^ g/L^*^	Buijs et al., 2014
Tackling tight regulations	Fatty alcohol (C16)	BY4741	Overexpression of Tyto alba FAR, *ACC1*, and *Y. lipolytica* ACL. Deletion of *RPD3*	1.1 g/L	Feng et al., 2014
	FAEE (N/A)	CEN.PK113	Abolishing Snf1-Dependent Regulation of ACC1 by introduction of two site mutations in ACC1, Ser659 and Ser1157	0.0158 g/L	Shi et al., 2014b
	FFA (C18)	BJ5464	Overexpression of ACC1^S1157A^	0.33 g/L	Choi and Lee, 2013
Improving resistance to toxic products	Alkanes (C9–C12)	BY4741	Transcriptome analyses and overexpressing of *YOR1, SNQ2, PDR5*, and *PDR15*	Intracellular C10 and C11alkanes amount was lowered by 33 and 94.4%, respectively	Ling et al., 2013
	Alkanes (C8–C12)	BY4741	Heterologous expression of ABC2 and ABC3 transporters	Increased the tolerance limit 80-fold against decane	Chen and Chang, 2013

* *Calculated based on the assumption that the final biomass was 5 g/L*.

## Bottlenecks of producing fatty acid-derived biofuels in yeast and their solutions

To produce fatty acid-derived biofuels, the fatty acid biosynthesis (FAB) pathway needs to be appropriately engineered. FAB begins with the conversion of acetyl-CoA into malonyl-CoA by acetyl-CoA carboxylase (ACC). Then these two precursors are condensed by fatty acid synthases (FASs) to fatty acids using the malonyl-CoA as the extender unit. Each elongation of two carbon unit in FAB costs 2 NADPH. In the cytosol of *S. cerevisiae*, FAB is catalyzed by type I FAS system, with all the functional domains organized into two subunits, encoded by *FAS1* (β-subunit) and *FAS2* (α-subunit) (Hiltunen et al., 2009; Chan and Vogel, 2010). Since all functional domains, including the ACP, are organized in the FAS complex, the whole FAB process is performed within the fatty acid elongation chamber after malonyl-CoA is loaded. The released fatty acyl-CoAs can be converted to the desired products, such as FFAs, fatty alcohols, and FAEEs by the corresponding terminal enzymes. However, *S. cerevisiae* naturally produces fatty acids at very low levels due to a series of obstacles (Lian and Zhao, 2014). Here in this review, we have summarized over 100 publications on metabolic engineering of yeast fatty acid metabolism and listed the bottlenecks into five categories: (1) the limited supply of FAB precursor; (2) the limited supply of cofactors; (3) the tight regulations of fatty acid metabolism; (4) the product toxicity; and (5) the lack of metabolic engineering tools in non-model oleaginous yeast.

### The limited supply of FAB precursors

In *S. cerevisiae*, the synthesis of fatty acids can take place in at least two subcellular compartments: cytoplasm (type I FAS) and mitochondria (type II FAS). The cytosol type I FAS produces most of the fatty acids in the cell, which indicates that the predominant efforts on metabolic engineering of fatty acid synthesis should be focused on improving the supply of cytosolic precursors (i.e., acetyl-CoA, malony-CoA, and fatty acyl-CoA) for fatty acid synthesis.

Acetyl-CoA is a key precursor in the biosynthesis of sterols, amino acids, fatty acids, and polyketides (Tai and Stephanopoulos, 2013; Lian et al., 2014; Shi and Tu, 2015). In *S. cerevisiae*, acetyl-CoA metabolism takes place in at least four subcellular compartments: nucleus, mitochondria, cytosol and peroxisomes. Since fatty acids are mostly synthesized in the cytosol of *S. cerevisiae*, the pyruvate-acetaldehyde-acetate pathway (PDC pathway) is one of the pathways used to produce acetyl-CoA for fatty acid synthesis (Chen et al., 2012). The cytosolic acetyl-CoA is tightly controlled (Pfeiffer and Morley, 2014), probably limiting in high glucose conditions (Postma et al., 1989; Chen et al., 2013) and separate from the other pools (Crabtree, 1928; Lian and Zhao, 2014), which is contrasted with bacterial acetyl-CoA (Takamura and Nomura, 1988; Cronan and Waldrop, 2002). The activation of acetate to acetyl-CoA was also found to be a rate-limiting step because of the feedback inhibition on ACS and the requirement of ATP input (Lian and Zhao, 2014).

Since the supplement of cytosol acetyl-CoA is very limited, the central metabolism of yeast should be engineered to redirect the metabolic flux to cytosolic acetyl-CoA biosynthesis (Krivoruchko et al., 2015). It has been found that deleting major ADHs in the cytosol (Δ*ADH1*–Δ*ADH4*), acetyl-CoA level was increased around 2-fold (Lian et al., 2014). In another study, 1.9-fold improvement in fatty acids production was achieved by knocking out *ADH1* in a fatty acid-producing host (Li et al., 2014). Beside engineering host metabolism, numerous heterologous pathways, including cytosolic pyruvate dehydrogenase (PDH) (Jing et al., 2011; Chen et al., 2013), pyruvate:formate lyase (PFL) (Kozak et al., 2014), pyruvate:ferrodoxin oxidoreductase (PFO), pyruvate: NADP^+^ oxidoreductase (PNO) (Inui et al., 1987), ATP-dependent citrate lyase (ACL), (Zaidi et al., 2012), acetylating aldehyde dehydrogenase (A-ALD), and phosphoketolase pathway (PK) (Sonderegger et al., 2004; de Jong et al., 2014), were introduced in *S. cerevisiae* to enhance the acetyl-CoA level in the cytosol, leading to the improvement of fatty acid production by 1.17-fold, n-butanol production by about 3-fold and FAEE production by 5.7-fold. In addition, the inactivation of acetyl-CoA consuming pathways could further increase the FAB activity. Glyoxylate shunt, which contributes to the transport and consumption of cytosolic acetyl-CoA in yeast, becomes the most important target (Chen et al., 2012; Tang et al., 2013). However, the combination of the “pull and push” engineering strategy failed to further increase the acetyl-CoA level. The unexpected results might be attributed to the accumulation of acetate to a cytotoxicity level that is accompanied with the decreased production of acetyl-CoA derived products (Lian et al., 2014).

Another key precursor in FAB, malonyl-CoA, is catalyzed from acetyl-CoA via acetyl-CoA carboxylase (ACC), which is encoded by the *ACC1* gene in *S. cerevisiae*. This is the first step in FAB and is well known as the rate limiting step (Li et al., 2014; Runguphan and Keasling, 2014; Zhou et al., 2014). Therefore, the pathways for malonyl-CoA synthesis need to be well engineered to improve the availability of malonyl-CoA in yeast. The most straightforward strategy is to overexpress the *ACC1* gene and *FAS* genes. When overexpressing all three FAB genes, *ACC1*, *FAS1*, and *FAS2* in *S. cerevisiae*, the production of FFAs achieved at a titer of approximately 400 mg/L, fatty alcohols at approximately 100 mg/L and FAEEs at approximately 5 mg/L directly from simple sugars (Runguphan and Keasling, 2014). Researchers also found that by introducing a heterologous FAS from *Brevibacterium ammoniagenes*, FAEE titer was increased by 6.3-fold compared to strains without expressing the heterologous FAS (Eriksen et al., 2015). In addition to the overexpression of *ACC1*, direct synthesis of malonyl-CoA from malonate by the malonyl-CoA synthetase (MCS) could also lead to improved malonyl-CoA levels. Basically, malonate was added exogenously and transported by a heterologous dicarboxylic acid plasma membrane transporter that was introduced to *S. cerevisiae* (Chen and Tan, 2013). The MCS from plants (Wang et al., 2014) or bacteria (Chen and Tan, 2013) has been cloned, characterized and functionally expressed in *S. cerevisiae*. Overexpression of a plant malonyl-CoA synthetase gene (*AAE13*) in *S. cerevisiae* resulted in 1.6-fold and 2.4-fold increases in lipid and resveratrol accumulation simultaneously (Wang et al., 2014). Considering the important role of malonyl-CoA as the precursor for the synthesis of a wide variety of value-added compounds (e.g., polyketides), malonate supplementation and MCS overexpression may be a possible route to improve cellular level of malonyl-CoA in yeast (Chen and Tan, 2013). However, in order to achieve the economic production of fatty acid-derived biofuels, the high cost of malonate (approximately $8000/ton) needs to be taken into consideration.

In addition to acetyl-CoA and malonyl-CoA, another important precursor for synthesizing fatty acid-derived biofuels is fatty acyl-CoA. To improve the fatty acyl-CoA level, the competing pathway, the β-oxidation cycle, was disrupted in *S. cerevisiae* by deleting POX1, a key enzyme in the β-oxidation pathway. Unfortunately, the disruption of β-oxidation cycle failed to improve the production of fatty alcohols and FAEEs (Runguphan and Keasling, 2014). However, by combining the strategy of disrupting the acyl-CoA transport to the β-oxidation pathway, avoiding TAG biosynthesis, and utilizing nitrogen limiting culture conditions to push carbon toward fatty acyl-CoA production, cytosolic acyl-CoA pools have been increased for FAEEs biosynthesis, leading to over 25 mg/L FAEEs produced, a 40% improvement over previous reports (Valle-Rodríguez et al., 2014).

### The limited supply of cofactors

**Figure 1:**
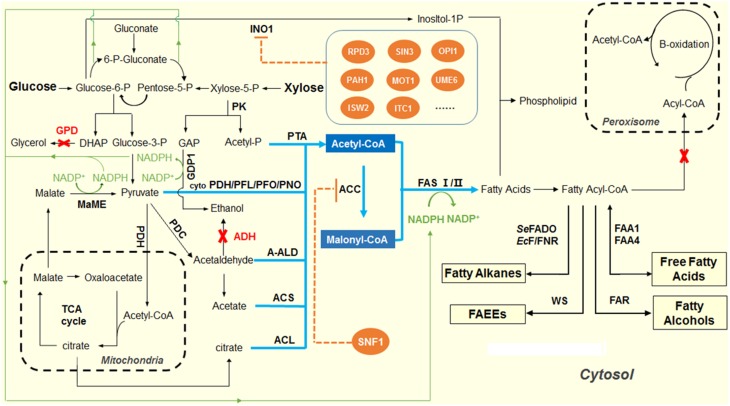
**Overview of the scheme for producing fatty acid-derived biofuels in yeast**. The pathways shown in blue indicate the design and construction of acetyl-CoA overproducing yeast strains. In *S. cerevisiae*, pyruvate dehydrogenase complex (PDH) was natively used to produce acetyl-CoA in mitochondria, while pyruvate decarboxylase (PDC) was used to produce acetyl-CoA in cytosol. The competing pathways, glycerophosphate dehydrogenase (GPD) for glycerol production and alcohol dehydrogenase (ADH) for ethanol formation, were inactivated to redirect the glycolytic fluxes to acetyl-CoA biosynthesis. Heterologous pathways, including cytosolic pyruvate dehydrogenase, PFL, pyruvate:formate lyase; PFO, pyruvate:ferrodoxin oxidoreductase; PNO, pyruvate:NADP^+^ oxidoreductase; engineered PDH-bypass pathway, ACL, ATP-dependent citrate lyase; A-ALD, acetylating aldehyde dehydrogenase; and PK, phosphoketolase pathway were introduced to enhance the acetyl-CoA level in the cytosol of yeast. The pathways shown in orange indicate the regulation of fatty acid biosynthesis by SNF1 that down-regulates ACC1 activity, and a cluster of repressors of inositol-1-phosphate synthase (INO1), in the phospholipid metabolism. The pathways shown in green indicate the efforts on improving the supply of NADPH by overexpressing the malic enzyme and NADP-GAP dehydrogenase (NADP-GAPDH) encoded by *GDP1*.

As shown in Figure 1, NADPH provides the reducing power during the FAB process. Generally, the elongation of the fatty acid will cost 2 NADPH to incorporate a C2 unit. In addition to fatty acid synthesis, NADPH is also involved in many other biological functions, such as lipid synthesis, cholesterol synthesis, and amino acid synthesis. In *S. cerevisiae*, the generation of NADPH occurs in cytosol, mitochondria and peroxisome. In the cytosol, the oxidative pentose phosphate pathway generates the majority of NADPH (Bruinenberg et al., 1983; Minard and McAlister-Henn, 2005). In mitochondria, the isocitrate dehydrogenase (IPD2) in the TCA cycle could generate NADPH. Peroxisomal isocitrate dehydrogenase (IPD3) catalyzes oxidation of isocitrate to alpha-ketoglutarate with the formation of NADPH, which is required for growth on unsaturated fatty acids (Henke et al., 1998; van Roermund et al., 1998). As one of the major NADPH-consuming processes, the FAB is greatly constrained by the lack of NADPH supply in the cytosol.

In order to improve the NADPH supply in the cytosol, different strategies have been adopted. For example, a non-phosphorylating, NADP^+^-dependent glycerol dehyde-3-phosphate dehydrogenase (GAPN), which catalyzes the irreversible oxidation of glyceraldehyde-3-phosphate and NADP^+^ into 3-phosphoglycerate and NADPH in glycolysis, was heterologously expressed to increase the NADPH supply and improved the productivity of fatty acid-derived biofuels (Guo et al., 2011). The overexpression of GAPN together with wax ester synthase (WS2) resulted in a final titer for FAEEs of 148 mg/L, a 40% increase compared to the control strain (de Jong et al., 2014; Shi et al., 2014a). In another study, overexpression of GAPN with enhanced malonyl-CoA supply led to a 70% increase in 3-hydroxypropionic acid production (Chen et al., 2014a). Another reported approach is the heterologous expression of GDP1, a NADP^+^-dependent glyceraldehyde-3-phosphate dehydrogenase (NADP-GAPDH, EC1.2.1.13). The GDP1 from *Kluyveromyces lactis* was expressed in *S. cerevisiae* strain together with the NADPH-consuming fungal xylose pathway, and increased the xylose utilization with a higher ethanol productivity and yield compared to the control strain without expressing GDP1 (Verho et al., 2003).

In addition to GAPN and NADP dependent GAPDH, another common strategy to achieve NADPH overproduction is to overexpress an NADP-dependent malic enzyme. Malate is mainly synthesized as an intermediate in the citric acid cycle. Malic Enzyme converts malate and NADP^+^ to pyruvate and NADPH, releasing one molecule of carbon dioxide. Overexpressing the malic enzyme from the oleaginous fungus *Mortierella alpina* in the fatty alcohol-producing strain leads to a marginal increase (about 14%) in the final fatty alcohol titer (Runguphan and Keasling, 2014). Due to the complicated generation and consumption pathways of NADPH in the metabolic network, new strategies need to be explored to increase the NADPH supply, which could further improve the productivity of fatty acid-derived biofuels.

### The tight regulations of fatty acid metabolism

Fatty acids are essential compounds in the cell that mainly serve a role as intermediates in lipid biosynthesis. Synthesis of fatty acids utilizes substantial amounts of metabolites, acetyl-CoA, ATP and NADPH, and may thus compete with other cellular processes dependent on these compounds. Due to the tight regulation of the FAS systems, *S. cerevisiae* does not naturally produce fatty acids to high levels to ensure proper fatty acid composition and homeostasis (Tehlivets et al., 2007). Fatty acid metabolism is regulated on both transcriptional and translational levels. As the key enzyme in FAB, the housekeeping enzyme FAS is expressed constitutively at a low level. It is found that some general yeast transcription factors (such as RAP1, ABF1, and REB1) (Schüller et al., 1994) and theinositol/choline-responsive transcription factor heterodimer (Ino2p and Ino4p) (Schüller et al., 1992) could activate its expression. Additionally, the FAS subunits are further regulated by proteolytic degradation of excess subunits. The intact FAS multimeric complex (alpha6-beta6) is stable, but its individual subunits are rapidly degraded (Egner et al., 1993). Under such tight regulations on multi-levels, the direct engineering of FAS was extremely difficult. Since only limited success was achieved in yeast to enhance fatty acid derived biofuel production via manipulating the structural genes, developing novel methods that can increase the flux toward producing fatty acid-derived biofuels becomes especially important. Two well-known families of transcriptional factors, SNF1 protein and the regulators of *INO1*, were focused in some of the recent reports since both families of the regulators could hamper the fatty acids synthesis via down-regulation of either ACC1 or phospholipid synthesis (Feng et al., 2014).

Straightforward overexpression of the *ACC1* gene can only improve the production of malonyl-CoA-derived molecules to a very limited extent. For example, less than 2-fold improvements were achieved in producing polyketide 6-MSA, FAEEs and 3-hydroxypropionic acid, by exclusively overexpressing *ACC1* (Wattanachaisaereekul et al., 2008; Shi et al., 2014b). Such results indicated the control of ACC1 enzyme activities is more complicated than gene overexpression, making it necessary to engineer the regulatory machinery. The ACC1 activity in *S. cerevisiae* is post-translationally regulated and repressed by various regulators. One of the most well-known regulators of ACC1 is SNF1, which not only represses the ACC1 activity, but also induces genes involved in gluconeogenesis, glyoxylate cycle, oxidation of fatty acids, as well as genes involved in the general stress response, pseudohyphal growth, aging, and ion homeostasis (Hong and Nielsen, 2012). The down-regulation of ACC1 activities by SNF1 could be eliminated by changing the phosphorylation recognition motif (Hyd-X-Arg-XX-Ser-XXX-Hyd) of SNF1. By mutating the putative phosphorylation sites from serine to alanine (ACC1^S659A,S1157A^), the ACC1 activity was increased by more than 3-fold and the production of malonyl-CoA derived metabolites, FAEEs and 3-HP, was increased by about 3.3- and 3.4-fold, respectively (Shi et al., 2014b).

The regulators of *INO1*, on the other hand, have been well known to transcriptionally control phospholipid synthesis by binding to *cis*-regulatory element UAS_INO_ in the promoter region of the *INO1* gene, which encodes inositol-1-phosphatesynthase as the first step for synthesis of inositol phosphates and inositol-containing phospholipids (Ambroziak and Henry, 1994; Feng et al., 2014). Since phospholipid synthesis requires fatty acids as precursors, it was expected that the deletion of negative regulators of phospholipid metabolism would enhance phospholipid production, and hence increase fatty acid synthesis. The family of regulators of *INO1* includes a dozen transcription factors that interact with each other to control the expression level of the *INO1* gene (Feng et al., 2014). In one of the recent studies, PAH1, RPD3, SIN3, OPI1, UME6, ITC1, ISW2, and MOT1 were chosen as the target regulators to knockout, since all of these transcription factors can decrease *INO1* expression in phospholipid production. For Δ*PAH1*, Δ*RPD3*, Δ*MOT1*, and Δ*OPI1*, the production of the target chemical, 1-hexadecanol, was enhanced by 60–170%, compared to the strain without knocking out the regulators. The highest titer was achieved at 122 mg/L in the Δ*RPD3* strain (Feng et al., 2014).

### The product toxicity

Hydrocarbons (e.g., alkanes) have been recently considered important next-generation biofuels. Several studies have demonstrated microbial production of alkane biofuels in both *E. coli* and yeast (Bernard et al., 2012; Choi and Lee, 2013; Buijs et al., 2014). Despite the promising potential of microbial alkane production, the yields and titers are key considerations for industrial-scale production. The toxicity of alkanes to microbial hosts can be a possible bottleneck for high productivity of alkane biofuels. Many studies have focused on finding the mechanism of the toxicity of fatty alkanes. It has been found that alkane products interact preferentially with cytoplasmic membrane, therefore disorganizing its structural integrity (Gill and Ratledge, 1972; Chen and Chang, 2013). Disruption of membrane structure impairs vital functions, such as the loss of ions, metabolites, lipids, and proteins, and the dissipation of the pH gradient and electrical potential. To overcome this toxicity issue, a clear understanding of the molecular mechanisms of interaction between yeast and alkanes could help to develop engineering strategies to improve microbial tolerance against alkane biofuel.

To tackle this toxicity issue caused by hydrocarbon biofuels, especially by alkanes, transcriptome analyses of *S. cerevisiae* have been accomplished, and suggested that C9 and C10 alkanes induced a wide range of rewiring of cellular metabolism, such as induction of efflux pumps, membrane modification, radical detoxification and energy supply (Ling et al., 2013). Since efflux pumps could possibly facilitate alkane secretion, and hence reduce the cytotoxicity, several studies have engineered the efflux pumps in *S. cerevisiae*. It is demonstrated that efflux pumps SNQ2P and PDR5P could reduce the intracellular levels of C10 and C11 alkanes and enhance the strain tolerance to alkanes. Upon 24 h exposure to C10 and C11, the amount of intracellular alkanes was lowered by 33 and 94.4%, respectively (Ling et al., 2013). In addition, heterologous expression of ABC2 and ABC3 transporters from *Y. lipolytica*, which could utilize alkanes as the sole carbon source, significantly increased tolerance of *S. cerevisiae* against decane and undecane through maintaining lower intracellular alkane level. In particular, ABC2 transporter increased the tolerance limit of *S. cerevisiae* about 80-fold against decane (Chen and Chang, 2013).

### The lack of metabolic engineering tools in non-model oleaginous yeast

Besides *S. cerevisiae*, some oleaginous yeast strains have the ability to accumulate lipids to high levels, up to more than 20% of their biomass (Beopoulos et al., 2009; Munch et al., 2015). Among these oleaginous yeasts, *Y. lipolytica* is a unique host for biochemical production due to its abilities to accumulate high levels of lipids and utilize hydrophobic and waste carbon sources. To this end, *Y. lipolytica* has attracted great attention as a potential biofuel producing host. In the past decade, a series of genetic tools have been developed to transform plasmids, knock out genes, and develop both episomal and integrative expression cassettes to enable metabolic engineering approaches in *Y. lipolytica*. However, compared to the model yeast strain such as *S. cerevisiae*, several functions have not yet been achieved. For example, the tunable and high-level gene expression tools are still lacking in *Y. lipolytica* (Blazeck et al., 2014). Overall, the lack of molecular biology tools for tunable, modular, and high-throughput modification of genetic parts limits the use of *Y. lipolytica* for producing fatty acid-derived biofuels.

To this end, prior attempts have focused on designing strong constitutive promoters in *Y. lipolytica* by modifying the expression range of endogenous promoters through point mutations. For example, error-prone PCR of the native *S. cerevisiae* TEF promoter yielded a library of mutant promoters with a nearly 17-fold range in relative expression levels (Blazeck et al., 2013). Following this research, a hybrid promoter approach was developed to produce a novel library of high-expressing, tunable promoters in *Y. lipolytica*. The result revealed promoters in *Y. lipolytica* were enhancer limited, and such limitation can be partially or fully alleviated through the addition of tandem copies of upstream activation sequences (UASs) (Blazeck et al., 2011). Other genetic tools, such as CRISPR and one-step recombination of multiple genes (Gao et al., 2014), are also being developed for *Y. lipolytica*. All of these rational metabolic engineering efforts could significantly enhance the lipogenesis titers in *Y. lipolytica*. However, the resulting strain still suffered from decreased biomass generation rates. Recently, a rapid evolutionary metabolic engineering approach linked with a floating cell enrichment process was employed to improve lipogenesis rates, titers, and yields. Through this iterative process, researchers were able to ultimately improve yields from their prior strain by 55% to achieve production titers of 39.1 g/L (Liu et al., 2015b). The highest lipid titer, 55 g/L, was achieved recently by simultaneous overexpression of delta-9 stearoyl-CoA desaturase (SCD), acetyl-CoA carboxylase (ACC1), and diacylglyceride acyl-transferase (DGA1) in *Y. lipolytica* (Qiao et al., 2015).

## Other potential issues in metabolic engineering of yeast to produce fatty acid-derived biofuels

### Difference of fatty acid metabolism between *E. coli* and *S. cerevisiae*

Beside *S. cerevisiae* and *Y. lipolytica*, the prokaryote model microorganism *E. coli* is another widely used industrial workhorse for producing fatty acid-derived biofuels. *E. coli* uses the type II FASs to catalyze FAB. The direct product of type II FASs is in the form of fatty acyl-ACPs, which need to be hydrolyzed by a thioesterase (TE) to release FFAs, and subsequently activated to fatty acyl-CoA by a fatty acyl-CoA ligase. Since this type of FASs was constructed by a series of mono-functional proteins that are discretely expressed from a series of separate genes, engineering on these structural genes was much easier than type I FASs. It is shown that when simply overexpressing the acyl-ACP thioesterase gene from *R. communis* in *E. coli*, the accumulation of FFAs could reach a high level of more than 2.0 g/L at 48 h, which was about 40-fold compared to the control strain (Zhang et al., 2011b). In comparison, by replacing the native promoters of *ACC1*, *FAS1*, and *FAS2* with strong and constitutive promoters in *S. cerevisiae*, their expression levels were increased about 7–16 fold, but the production of FFAs, fatty alcohols, and FAEEs were only increased by about 11-, 2-, and 4-fold, respectively, which is much lower than the improvement in *E. coli* (Lian and Zhao, 2014; Runguphan and Keasling, 2014). Therefore, the same strategy that works for *E. coli* may not work as well for yeast. Another example is the different behavior between the two species when engineering the β-oxidation pathway. A study in deletion of *POX1*, the first gene in the β-oxidation pathway in *S. cerevisiae*, did not improve fatty alcohol production titer (Runguphan and Keasling, 2014). But in combination with the deletion of other genes in the fatty acid degradation pathway, FFAs have been produced at high titers (8.6 g/L) in *E. coli* (Xu et al., 2013). Such discrepancy could probably result from the more complex regulatory mechanisms and cellular compartmentalization in *S. cerevisiae*.

### Balancing metabolic pathways in yeast

The strategies for balancing the enzyme activities and expression to tune metabolic flux are important to improve the productivity. In *E. coli*, a dynamic sensor-regulator system (DSRS) to produce fatty acid-based products has been established, and demonstrated its use for biodiesel production (Zhang et al., 2012). By incorporating the *Bacillus subtilis* trans-regulatory protein fapR and the *cis*-regulatory element fapO, a malonyl-CoA responsive sensor has been developed which holds great promise in overcoming critical pathway limitations and optimizing titers and yields of target products (Xu et al., 2014). These sensors reported in *E. coli* could potentially control and optimize carbon flux, which leads to robust biosynthetic pathways. However, the strategy of balancing metabolic pathways has not yet been fully explored in yeast strains such as *S. cerevisiae*. The challenge in building a regulatory system in yeast is the lack of engineered promoters and functionally expression genetic parts under the tightly regulated genetic background, since the DSRS requires the combination of various kinds of promoters that can both detect key intermediates in the synthesis cascade and control gene expression to improve production of the desired chemicals. To establish a similar DSRS in yeast, the frontier of eukaryotic promoters needs to be further explored to develop new systems such as RNA based response and regulatory elements.

### Optimizing fermentation conditions to further improve the productivity

Process optimization, aiming at maintaining optimal and homogenous reaction conditions, minimizing microbial stress exposure, and enhancing metabolic activities, plays vital roles in achieving high productivity of fatty acid-derived biofuels. By identifying the most relevant process parameters that affect product yield and quality, the fermentation strategy could be optimized and maximize the productivity of a metabolically engineered strain. Recently, the importance of optimizing fermentation conditions has been demonstrated. In a research project related to the enhancement of FAEE production in *S. cerevisiae*, nitrogen limiting culture was used to achieve an immediate increase (over 3.5-fold) in the titer of FAEEs compared to the control culture conditions (Thompson and Trinh, 2014). In addition, switching from the batch mode to fed-batch, resting cell fermentation mode, the highest titer of 1-hexadecanol produced by an engineered *S. cerevisiae* strain could reach 1111 mg/L, a 10-fold increase compared to the previous report on engineering yeast for fatty alcohol production (Hong and Nielsen, 2012). However, the systemic characterization and improvement of fermentation conditions are still required for improving the biofuel productivity, especially for pilot scale production.

## Concluding remarks

To engineer yeast for production of a wide range of fatty acid-derived biofuels, bottlenecks such as the limited supply of FAB precursor and cofactors, the tight regulations of fatty acid metabolism, and the product toxicity to host cells needs to be overcome to achieve high productivity. In order to tackle these limitations, extensive efforts on metabolic engineering have been made to improve the productivity of fatty acid-derived biofuels in yeast strains. Until now, most of the researches focused on the improvement of precursors and co-factor supply in FAB. After engineering both the heterologous and endogenous pathways, significant increase of the productivity of fatty acids-derived chemicals has been achieved. There is also an interesting trend of engineering the regulatory elements of yeast metabolism to relieve the tight regulation on some rate limiting enzymes and improve the productivity. In addition to all of these successful strategies, there are other possible approaches that could also help to improve the yeast-based production of fatty acid-derived biofuels, such as the design of dynamic *in vivo* sensor system. To date, no single synthetic biology approach could completely meet the demands to develop an almighty host for producing fatty acid-derived biofuels. Therefore, the integration of “omics” analysis [e.g., genomics (Cherry et al., 2012; Lee et al., 2013), transcriptomics (Wang et al., 2009; Rossouw et al., 2010), proteomics (Chen and Snyder, 2010; Rossouw et al., 2010), metabolomics (Smedsgaard and Nielsen, 2005; Jewison et al., 2012), and fluxomics (Feng and Zhao, 2013; Winter and Kromer, 2013)] with the rational applications of appropriate biomolecular engineering toolbox could make steady progress toward the goal of engineering yeast to be a more suitable host for producing fatty acid-derived biofuels.

### Conflict of interest statement

The authors declare that the research was conducted in the absence of any commercial or financial relationships that could be construed as a potential conflict of interest.
